# Associations between dietary micronutrient intake and molecular-Bacterial Vaginosis

**DOI:** 10.1186/s12978-019-0814-6

**Published:** 2019-10-22

**Authors:** Susan Tuddenham, Khalil G. Ghanem, Laura E. Caulfield, Alisha J. Rovner, Courtney Robinson, Rupak Shivakoti, Ryan Miller, Anne Burke, Catherine Murphy, Jacques Ravel, Rebecca M. Brotman

**Affiliations:** 1 Department of Medicine, Johns Hopkins University School of Medicine, 5200 Eastern Ave, MFL Center Tower, Suite 381, Baltimore, MD 21224 USA; 2 Center for Human Nutrition, Johns Hopkins Bloomberg School of Public Health, Baltimore, MD USA; 3 Department of Behavioral Health and Nutrition, University of Delaware, Newark, DE USA; 4 Institute for Genome Sciences, University of Maryland School of Medicine, Baltimore, MD USA; 5 Department of Epidemiology, Columbia University Mailman School of Public Health, New York, NY USA; 6 Department of Pediatrics, University of Maryland School of Medicine, Baltimore, MD USA; 7 Department of Obstetrics and Gynecology, Johns Hopkins University School of Medicine, Baltimore, MD USA; 8 New York Medical College, Maria Fareri Children’s Hospital, Valhalla, NY USA

**Keywords:** Bacterial Vaginosis, Vaginal microbiome, Betaine, Food frequency questionnaire

## Abstract

**Objectives:**

Bacterial vaginosis (BV), a clinical condition characterized by decreased vaginal *Lactobacillus* spp., is difficult to treat. We examined associations between micronutrient intake and a low-*Lactobacillus* vaginal microbiota as assessed by molecular methods (termed “molecular-BV”).

**Methods:**

This cross-sectional analysis utilized data collected at the baseline visit of the Hormonal Contraception Longitudinal Study, a cohort of reproductive-aged women followed over 2 years while initiating or ceasing hormonal contraception (HC). The Block Brief 2000 Food Frequency Questionnaire was administered and micronutrient intakes were ranked. Vaginal microbiota composition was assessed using 16S rRNA gene amplicon sequencing and clustered into community state types (CSTs) based on the types and relative abundance of bacteria detected. Associations between the lowest estimated quartile intake of nutrients and having a low-*Lactobacillus* CST (molecular-BV) were evaluated by logistic regression. Separate models were built for each nutrient controlling for age, body mass index, behavioral factors, HC use and total energy intake. We also conducted a literature review of existing data on associations between micronutrient intakes and BV.

**Results:**

Samples from 104 women were included in this analysis. Their mean age was 25.8 years (SD 4.3), 29.8% were African American, 48.1% were using HC, and 25% had molecular-BV. In adjusted multivariable analyses, the lowest quartile of betaine intake was associated with an increased odds of molecular-BV (aOR 9.2, *p* value < 0.01, [CI 2.4–35.0]).

**Conclusions:**

This is the first study to assess the association between estimated micronutrient intake and molecular-BV. Lower energy-adjusted intake of betaine was associated with an increased risk of molecular-BV. Betaine might have direct effects on the vaginal microenvironment or may be mediated through the gut microbiota. Additional research is needed to determine reproducibility of this finding and whether improved intake of select micronutrients such as betaine decreases the risk of BV and its sequelae.

## Plain English summary

Bacterial vaginosis (BV) is a common cause of vaginal complaints in women of reproductive age. Unfortunately, it is difficult to treat and frequently recurs. If low micronutrient intake contributes to BV, then dietary supplements or diet-based interventions might be a way to help treat and prevent BV. Previous studies that examined the relationship between micronutrient deficiencies or low dietary intake and BV have yielded conflicting results and were based on BV evaluation by microscopy or clinical criteria. Here we utilized 16S rRNA gene amplicon sequencing to characterize the communities of bacteria living in the vagina and found an increased risk of a low-*Lactobacillus* vaginal microbiota (or “molecular-BV”) among women with the lowest quartile of the micronutrient betaine. Larger studies will be needed to determine whether improving intake of betaine could help treat or prevent BV.

## Background

The clinical condition of bacterial vaginosis (BV), is characterized by low levels of *Lactobacillus* spp*.* and higher abundances of gram negative and anaerobic bacteria [1]. BV is a leading cause of vaginal complaints in women of reproductive age. Importantly, low-*Lactobacillus* vaginal microbiota have been associated with an increased risk for acquisition of sexually transmitted infections, including HIV [2–4].

BV is diagnosed in clinical settings by the Amsel’s criteria (i.e. having at least three out of four of the following: thin, homogenous vaginal discharge, pH > 4.5, 20% of clue cells on saline microscopy, and a fishy odor after addition of 10% potassium hydroxide to a slide of secretions (whiff test)). Historically, in research settings, BV has been assessed by Gram’s stain of vaginal secretions (Nugent score) [1]. Recently, 16S rRNA gene amplicon sequencing techniques have enabled a higher resolution understanding of the bacterial composition of communities that inhabit the vagina, and have enabled the identification of several clusters, termed “community state types”(CSTs), that differ based on the composition and relative abundance of bacterial taxa. The CSTs are dominated by different species of *Lactobacillus*, or are characterized by a paucity of *Lactobacillus* spp. The latter CSTs are comprised of a variety of anaerobes such as *Gardnerella vaginalis* and *Sneathia spp.*, and are consistent with BV [5]. The low-*Lactobacillus* CSTs have been collectively termed “molecular-BV,” as they reflect similar low-*Lactobacillus* states that are captured by Nugent score and Amsel’s criteria [6].

BV is difficult to treat, with nearly 60% of women experiencing recurrence within 12 months after antibiotics [7]. Therefore, identifiable dietary risk factors for BV would be of interest as potential therapeutic targets. Several studies have reported associations between BV and increased or decreased serum concentrations of nutrients including vitamins D, A, C, E, iron or β-carotene and dietary or supplemented intake of vitamin A, E, folate, calcium, β-carotene or iron, but results have been inconsistent (See Additional file 1: Table S1) [8–24]. Furthermore, studies have not examined relationships between dietary intake of nutrients and the vaginal microbiota as assessed by molecular tools. Most prior studies have been based on assessment of vaginal smears or clinical diagnosis of bacterial vaginosis. Only one study examined associations between iron supplementation and CST [10]. We conducted a cross-sectional analysis of associations between dietary micronutrient intake and molecular-BV among women of reproductive age. We also conducted a literature review to summarize evidence relating nutrient intake and BV (Additional file 1: Table S1).

## Methods

### Study setting

We analyzed the baseline visit of the Hormonal Contraception Longitudinal Study, a cohort of reproductive-aged women in Baltimore, MD, recruited between the years 2011–2015 who reported at enrollment an intention to initiate or cease hormonal contraception (HC). Clinicians collected mid-vaginal Eswabs (Copan Diagnostics, Murrieta, CA) which were stored at − 80 °C in 1 ml Amies transport medium for 16S rRNA gene sequencing. Participants filled out a detailed behavioral questionnaire at enrollment, and also completed a Block Brief 2000 Food Frequency Questionnaire (FFQ) [25]. This short questionnaire takes about 15–20 min to complete, and is designed to rank individuals along the distribution of dietary nutrient intake. It asks participants to estimate intake of specific foods over a year and provides estimates of average daily intake of micro and macronutrients (See Additional file 2: Figure S1 for a list of diet analysis output variables produced by the questionnaire). The food list for this questionnaire was developed from the NHANES III dietary recall data and the nutrient database was developed from the USDA Nutrient Database for Standard Reference. All study participants provided informed consent and this study was approved by the institutional review boards at the Johns Hopkins School of Medicine and the University of Maryland Baltimore.

### Vaginal microbiota characterization

All vaginal Eswabs (*n* = 104) were first extracted with the QS DSP Virus/Pathogen Midi Kit (Qiagen) on the QiaSymphony platform. Three samples were reprocessed with the MagAttract Microbial DNA Kit (Qiagen) using a custom automated protocol on the Hamilton Microlab Star because the samples resulted in less than< 15,000 reads with the first round of sequencing. Bead disruption and complete lysis are similar in both DNA extraction approaches. For the QiaSymphony kit, Eswabs in Amies samples were thawed on ice and 500 μl was used as input [26], while for the MagAttract kit, 200 μl was used. For both kits, the manufacturer protocols were followed. Cells were lysed on a TissueLyser instrument (Qiagen) at 20 Hz for 20 min, and DNA was eluted in final volume of 110 μl. Water was processed in parallel with samples through the DNA extraction process and added as template during the first round of PCR. These acted as quality control for the PCR steps, so if a band was detected in negative controls, the PCR would be redone. If bands persisted in the second PCR, samples on that plate would be re-extracted to try to eliminate the contamination. Negative controls were not used here to remove any taxa from analysis.

**Fig. 1:**
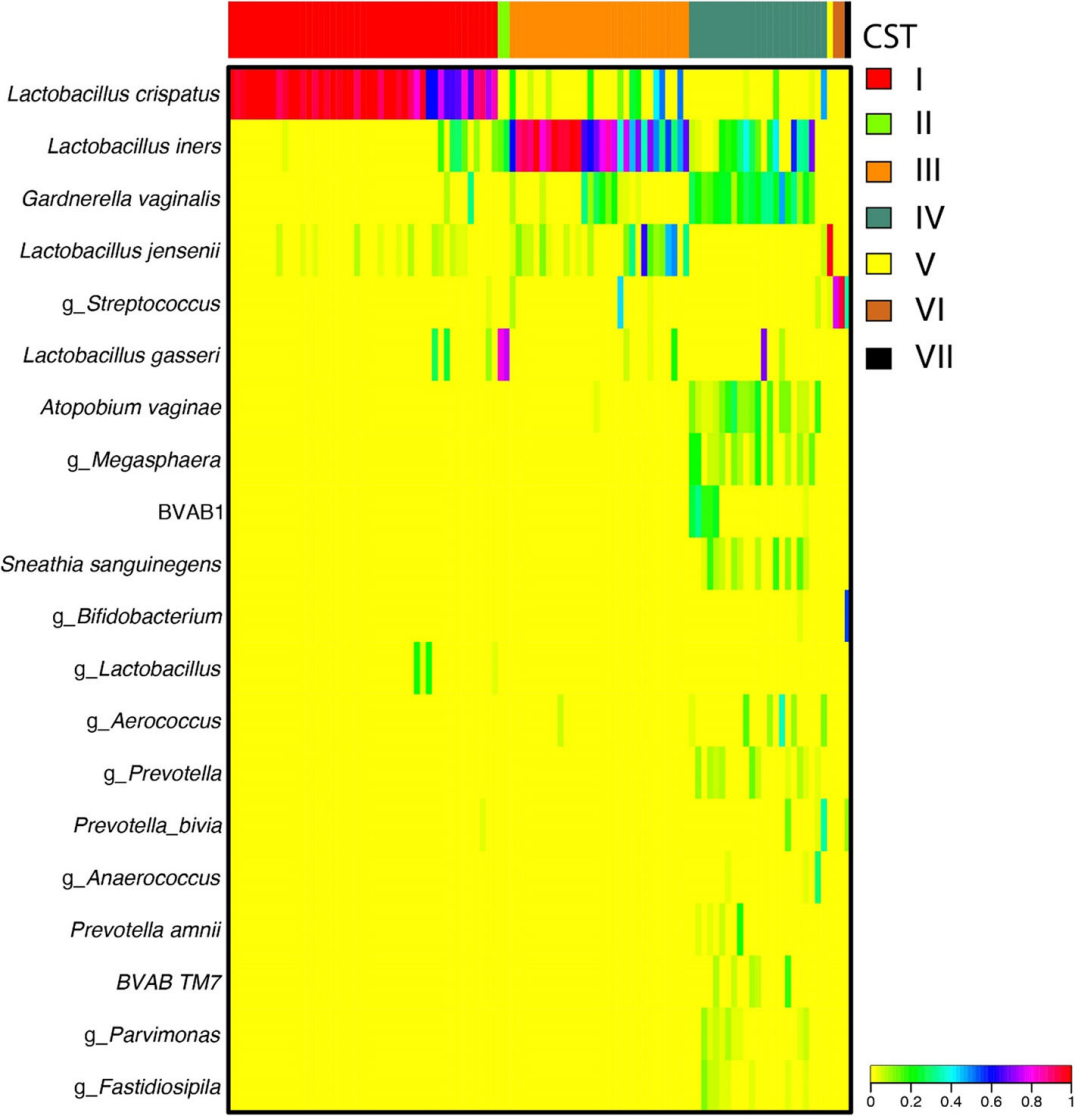
Heatmap of 104 vaginal microbiota, grouped into Community State Types (CSTs) based on bacterial composition and relative abundance

The vaginal microbiota was characterized by sequencing the V3-V4 regions of the 16S rRNA gene. Library construction was performed using a 2-step PCR protocol, sequencing was carried out on the Illumina HiSeq 2500 platform using Rapid Run Chemistry,as described previously [26]. The raw sequence data was processed using DADA2 [27], and amplicon sequence variants (ASVs) were classified taxonomically at the genus level using the RDP Naïve Bayesian Classifier [28] trained with the SILVA v128 16S rRNA gene sequence database [26, 29]. ASVs of major vaginal taxa were further speciated using speciateIT (http://ravel-lab.org/speciateit/). Taxa were only removed if they were less abundant than 10^–5.5^ abundance across all 4479 samples in the Parent study, and samples with fewer than 5000 reads were not included in the analysis. Samples included in this analysis had a median of 55,162 and a mean of 59,110 sequences (range of 17,313-235,834). Taxonomic data from a pool of 4479 urine and vaginal samples from the Hormonal Contraception Longitudinal Study were included in the clustering process to assign community state types (CSTs), however the study is restricted to the 104 baseline samples. Hierarchical clustering based on Jensen-Shannon distances between samples and Ward linkage was used to determine clusters for CST assignment. CSTs were characterized by dominance of the following bacteria (See Fig. 1): CST I – *L. crispatus*, CST II – *L. gasseri*, CST III – *L. iners*, CST IV – diverse anaerobes, CST V – *L. jensenii*, CST VI – *Streptococcus* spp., CST VII – *Bifidobacerium* spp. For the analysis herein, CSTs were collapsed into those dominated by *Lactobacillus spp.* (CST I, II, III, V) and those with low or no *Lactobacillus spp.* (CST IV, VI, and VII), termed “molecular-BV” [6]. Additional file 1: Table S2 includes details of the relative abundance of major taxa in each CST.

### Statistical analysis

Subjects with implausible energy intakes (of < 500 or > 3500 kcal) were excluded. A binary variable was created classifying women as having molecular-BV (i.e. low-*Lactobacillus* vaginal microbiota: CSTs IV, VI and VII) or not. Sample size was not sufficient for multinomial modeling to differentiate nutrients associated with each of the seven CSTs. We assessed intakes of all micronutrients listed in Additional file 2: Figure S1, including vitamins, minerals, methyl donors, carotenoid-derived antioxidants, essential fatty acids, and selected phytoestrogens. A binary variable was created based on the lowest intake quartile for a given nutrient versus the three higher quartiles. Associations between demographic, behavioral and clinical factors and molecular-BV were analyzed with student’s t-test and chi-squared tests. Associations between nutrient intake and molecular-BV were analyzed by logistic regression. Separate models were built for each nutrient adjusting for usual total energy intake, as well as potential confounding factors, such as age, body mass index (BMI = weight (kg)/height (m)^2^) number of male partners in the last 2 months and current hormonal contraception use. We first explored dietary intake with each nutrient intake divided into quartiles. Based on these analyses, we dichotomized nutrient intake. Some *Lactobacillus iners*-dominated vaginal microbiota have, (similar to low-*Lactobacillus* vaginal microbiota), been associated with increased risk for BV (and it is often the first species to expand after BV treatment [30],). *L. iners*-dominated vaginal microbiota have also been associated with STI acquisition and other poor reproductive outcomes [31, 32]. Therefore, we conducted an additional sensitivity analysis in which 3-category variables were created classifying women as having: 1.molecular BV (i.e. low-*Lactobacillus* CSTs IV, VI, VII), 2. *L. iners*-dominated (CST III) or 3. all other *Lactobacillus-*dominated CSTs. We then built multinomial models to assess how nutrients were associated with these categories, again adjusting for energy intake and potential confounding factors. All analyses were conducted using STATA v14 (StataCorp LLC, College Station, TX).

### Literature review

Please see supplementary methods and Additional file 1: Table S1 for search methods and results.

## Results

After eliminating patients with very low (*n* = 4, (3.6%)) or high (n = 4, (3.6%)) estimated energy intakes, data from 104 female subjects were available for analysis. The majority (60%) of patients were Caucasian, and 30% were African American (See Table 1). Patients’ mean age was 26, and nearly 50% were using HC at the time of entry into the study. African-American patients had a higher mean BMI (mean 34.7, Standard Deviation (SD) 7.8) than Caucasian (mean 24.7, SD 5.2) or patients of other ethnicity categories (mean 27.0, SD 10.1), *p* < 0.01). Patients with molecular-BV had a higher BMI (p < 0.01), were more likely to engage in vaginal douching (p < 0.01) and were less likely to be using HC at study entry (*p* = 0.01) compared to those with *Lactobacillus*-dominated CSTs (See Table 1).

**Table 1:** Demographic characteristics of subjects in study

	Overall *N* = 104	*Lactobacillus-*dominated *N* = 78	Molecular-BV *N* = 26	*p*-value
Age mean (SD)	25.8 (4.3)	25.9 (4.2)	25.7 (4.6)	0.83
Race				
White	60 (57.7)	49 (62.8)	11 (42.3)	0.10
Black	31 (29.8)	19 (24.4)	12 (46.2)	
Other	13 (12.5)	10 (12.8)	3 (11.5)	
Body mass index mean (SD)	28.0 (8.0)	26.6 (7.2)	32.2 (9.1)	<0.01
Smoking, last 2 months	12 (11.5)	8 (10.3)	4 (15.5)	0.48
Current douching	7 (6.7)	1 (1.3)	6 (23.1)	<0.01
# Male partners, last 2 months				
0	19 (18.3)	16 (20.5)	3 (11.5)	0.17
1	81 (77.9)	59 (75.6)	22 (84.6)	
2	3 (2.9)	3 (3.9)	0 (0.0)	
3	1 (1.0)	0 (0.0)	1 (3.9)	
Current hormonal				
contraception use	50 (48.1)	43 (55.1)	7 (26.9)	0.01

In analysis controlling only for total energy intake, having the lowest quartile intake of a number of nutrients was statistically significantly associated with decreased odds of molecular-BV as compared to those with higher intakes. (Table 2 lists odds ratios for nutrients found in bivariable analysis controlling only for total energy intake and a *p* < 0.10. We also reported on calcium and vitamin C as these were linked in the literature to BV). After adjustment for confounding factors (see footnote Table 2), participants with the lowest quartile intake of betaine had a statistically significantly increased odds of molecular-BV (aOR 9.2, *p* < 0.01, (95% CI: 2.4–35.0)). We conducted additional analyses in which we also included menses in the last week and antibiotic use in the last 30 days in our models, however this did not substantially alter our results, and we present the more parsimonious model in Table 2.

**Table 2:** Associations between usual micronutrient intakes and molecular-BV: bivariable and multivariable models analyzing the lowest versus the top three nutrient quartiles (reference)

Model 1		Model 2		
Micronutrient^a^	aOR (CI)^b^	p	aOR^c^	p
Betaine	9.1 (2.8–29.7)	<0.01	9.2 (2.4–35.0)	<0.01
Selenium	4.7 (1.3–16.6)	0.02	2.7 (0.7–10.6)	0.15
Zinc	5.0 (1.4–18.1)	0.01	3.0 (0.7–12.4)	0.13
Magnesium	3.1 (0.9–10.4)	0.07	1.6 (0.4–6.9)	0.50
Folate	2.8 (0.9–8.9)	0.09	1.7 (0.5–6.4)	0.41
Vitamin D	2.4 (0.8–7.2)	0.11	1.4 (0.4–4.9)	0.58
Vitamin A (RE)	2.8 (1.0–7.8)	0.05	2.7 (0.8–8.8)	0.10
Iron	3.1 (0.9–10.6)	0.07	1.7 (0.4–6.7)	0.48
Vitamin E	3.2 (0.9–11.2)	0.07	2.2 (0.56–9.0)	0.26
Vitamin C	1.7 (0.6–5.1)	0.32	1.4 (0.4–4.8)	0.58
Calcium	1.3 (0.4–4.2)	0.62	0.8 (0.2–2.8)	0.67
Beta-Carotene	2.1 (0.8–5.7)	0.15	1.9 (0.6–6.1)	0.28
Niacin	3.3 (0.9–11.9)	0.06	1.8 (0.4–7.2)	0.43
Thiamin	3.3 (0.9–11.9)	0.06	2.3 (0.6–9.3)	0.25
B6	3.3 (0.9–11.8)	0.06	1.8 (0.4–7.6)	0.43
Lutein	2.6 (1.0–7.2)	0.06	2.6 (0.85–7.9)	0.10

^a^First quartile; reference = top 3 quartiles. ^b^ Adjusted for energy intake ^c^ Adjusted for age, BMI, number of male sexual partners in last 2 months, hormonal contraception and energy intake

Results were consistent in sensitivity analysis where the outcome was a categorical variable with three categories: 1: combined high-*Lactobacillus* CSTs I, II, V as the reference, 2: the *L. iners*-dominated CST III and 3: molecular-BV. There were no statistically significant associations between lowest quartile nutrient intakes and CST III, however, having the lowest quartile of betaine intake was associated with an increased risk of having molecular-BV (RRR 11.2, *p* < 0.01, [CI: 2.5–50.1]). In a further sensitivity analysis, removing the three samples which were in CST VI and VII (as they were low in *Lactobacilli* but not dominated by classically BV-associated bacteria) from analysis, results were again consistent with increased odds of molecular-BV in the lowest quartile intake of betaine versus higher quartiles (aOR 6.9, *p* value < 0.01, [CI: 1.7–28.3]). Zinc and selenium were highly significant in bivariable analysis but were of borderline significance in the full model (*p* ≤ 0.15). Vitamin A and lutein were also of borderline significance in the full model (*p* = 0.10).

## Discussion

In this study, we found those with the lowest quartile of energy adjusted intake of betaine were more likely to have molecular-BV. Based on our literature review (See Additional file 1: Table S1), most previous studies examining relationships between micronutrients and BV focused on serum vitamin D. However, study designs were heterogeneous, and results were conflicting. Five studies (1 longitudinal and 4 cross-sectional observational studies) showed an increased risk of BV with low vitamin D [9, 16–18, 20], but three studies (all cross-sectional observational studies) [8, 11, 14] suggested no association, or even a reverse association with low vitamin D. Two randomized controlled trials examined vitamin D supplementation. One showed some benefit in asymptomatic BV [12], while another showed no improvement with symptomatic BV recurrence in vitamin D supplementation [13]. These studies were all based on vaginal microbiota assessment by Nugent score or Amsel’s criteria for the diagnosis of BV. In our study, there was a point estimate toward increased risk of molecular-BV with low vitamin D intake, but the finding was not statistically significant (aOR:1.4 (95% CI: 0.4–4.9)). Importantly, our study adds to the literature by assessing relationships between intake of these micronutrients and vaginal microbiota as assessed by 16S rRNA gene amplicon sequencing—i.e. molecular-BV.

Fewer studies have examined other micronutrients. One study found that women with subclinical iron deficiency were more likely to have BV [23]. However, two other studies did not find a relationship between iron and BV via serum measures [21]. One study nested within an RCT of iron supplementation in Burkina Faso found that BV prevalence (as assessed by Nugent score and 16S rRNA gene sequencing) did not differ by iron supplementation group, and in fact at baseline those who were iron deficient were more likely to have *Lactobacillus*-dominated vaginal microbiota [10]. One study found a decreased prevalence of BV with increased serum vitamin A [21] and another found a decreased incidence of BV with vitamin A supplementation [19]. A third found a decreased prevalence of genital tract infections associated with low serum vitamin A [24]. Two studies showed an association between β-carotene supplementation [19] or decreased serum β-carotene concentrations and BV [21]. One also found an association with serum vitamin E concentration and BV [21]. Two studies showed an inverse association between serum folate and BV [18, 21]. One study reported an inverse relationship between measures of diet quality and BV [15].

Finally, one study published by Neggers et al. [22] assessed dietary intake in a sample of *N* = 1521 primarily lower socioeconomic status African-American women from Alabama. In that study, significant associations were found between severe BV (defined as Nugent score ≥ 9 and vaginal pH > 5) and low intakes of folate (aOR 0.4, CI: 0.2–0.8), vitamin E (aOR 0.4, 0.2–0.8) and calcium (aOR 0.4, CI: 0.3–0.7). Our study found relationships with a different micronutrient (betaine) than the ones identified in the study by Neggers et al. This may have been due to three main differences in study design and population. First, the Neggers et al. study had a much larger sample size and used the full FFQ rather than the brief FFQ. Second, the Neggers et al. study population was predominantly African-American, whereas ours was predominantly White. And third, the Neggers et al. study assessed outcomes with BV as diagnosed by Nugent score and severe BV as defined by Nugent score and pH, where we assess BV as defined by compositional analysis of the microbiota.

There are several plausible mechanisms for why betaine may be associated with BV. Betaine is a small zwitterionic compound found in plants, animals and microorganisms. Dietary sources of betaine include seafood (especially marine invertebrates), wheat germ or bran, and spinach; in mammals it can also be obtained by endogenous synthesis from choline [33]. Dietary betaine is quickly absorbed, primarily in the small intestine [34, 35]. Betaine’s principal physiologic role is as an osmolyte and methyl donor [35]. As an osmolyte, betaine protects cells, proteins, and enzymes from environmental stress, while as a methyl donor it serves important roles in hepatic, cardiovascular and renal health. It is possible that betaine serves an as yet unrecognized role in stabilizing and maintaining vaginal epithelial cell health and hence positively impacts the vaginal microbiota or the host-microbiota interaction. Alternatively, betaine may positively impact the growth of vaginal *Lactobacillus* spp*.* and the production of lactic acid (which lowers the vaginal pH and may protect against overgrowth of BV-associated bacteria). In vitro, betaine has been noted to play an important role in osmotolerance and survival of *Lactobacillus* species [36], and it has been shown to enhance production of L-lactic acid by *Lactobacillus* species [37]. Whether betaine plays a role in osmotolerance of BV associated bacteria is not known. Of note, betaine may also be metabolized to form the biogenic amine trimethylamine (TMA), which has been associated with BV symptoms, however an analysis did not show any differences in vaginal levels of betaine between CSTs, and it is unclear how dietary betaine might relate to TMA in the vagina [38].

It is also possible that the impact of betaine on the vaginal microbiota could be mediated through effects on the gut microbiota. Of note, several studies have noted concordance between rectal and vaginal carriage of specific bacteria, including *Lactobacillus* spp. [39–41] and rectal carriage of *Lactobacillus* spp. has been associated with decreased risk of BV [40]. In one study, conducted in rats, high levels of betaine supplementation improved the function of digestive enzymes and increased the relative abundance of the genus *Lactobacillus* in the gut microbiota of salt-stressed animals [33]. In a study in pigs, there was a trend towards increased *Lactobacillus spp.* (assessed via qPCR) in the stool of animals given a mixed dietary supplement of betaine, an organic acid blend and inulin, though interestingly animals supplemented with betaine alone had decreased *Lactobacillus* spp. [34] However, it is unclear how applicable this limited animal data may be to humans.

CST III, vaginal microbiota dominated by *L. iners*, may also represent a sub-optimal vaginal microbiota as it has been associated with increased risk of BV recurrence, STI acquisition and poor birth outcomes, similar to molecular-BV [31, 32]. However, in sensitivity analysis splitting CST III from the other *Lactobacillus*-dominated CSTs, there were no statistically significant associations between lowest quartile micronutrient intakes and CST III, though associations with molecular-BV and lowest quartile intake of betaine remained statistically significant.

Our study was innovative in that we related dietary measures to molecular-BV as measured by 16S rRNA gene sequencing. Importantly, molecular-BV presents a higher-resolution assessment of the vaginal microbiota than Amsel-BV or Nugent-BV [6]. However, it had several limitations. First, we had a relatively small sample size (*n* = 104), which limited the analyses that could be conducted. We could not determine associations other than molecular-BV vs. *Lactobacillus* dominated CSTs and to a lesser extent the *L. iners*-dominated CST III. Nor were we able to adjust for factors such as number of sexual partners, condom use, recent antibiotic use or menses. We were unable to correct for multiple comparisons in the analysis. Since the study was cross-sectional in design, we were not able to account for potential fluctuations in the vaginal microbiota or nutrition over time. It is well documented that the vaginal microbiota often fluctuate between CST III and CST IV [42], so there could have been some non-differential misclassification. However, we would expect that if there was non-differential misclassification, the odds ratio would have tended toward the null. Instead we observed a statistically significant point estimate, which suggests that the true risk, without the noise of misclassification, may be even stronger. Brief food FFQs, including the one that was used in this study to estimate micronutrient intake, do not cover the full list of foods as in the Full Length FFQs. The brief FFQ likely underestimates usual intake of energy and nutrients, and can only be used to rank nutrient intakes between women in this study. Although it was not possible to estimate absolute nutrient intake, adjustments for energy intake allowed us to evaluate risks associated with low nutrient intake in the diet. Lastly, only dietary intake was assessed, and it could have been beneficial to measure biochemical indicators of nutrients to determine if nutrient deficiencies existed.

## Conclusions

Emerging evidence suggests that higher quality diets may be associated with lower risk of BV. Effects on BV might be mediated through direct effects in the vagina or via an impact of diet or specific micronutrients on the gut microbiota. Findings from our study suggest a relationship between low intake of betaine and low-*Lactobacillus* vaginal microbiota (i.e. molecular-BV). Ultimately, interventions to improve dietary intake of betaine may hold promise as a way to ameliorate BV. However, further, larger studies which utilize more precise methods to measure betaine intake will be needed to verify and expand these results.

## Supplementary information

**Additional file 1: Table S1.** Literature Review: Micronutrients and Vaginal Dysbiosis. **Table S2.** Relative abundance of major taxa in each CST

**Additional file 2: Figure S1.** List of Main Diet Variables Produced by the Block Brief Questionnaire.

## Data Availability

The 16S rRNA gene amplicon data will be released in NCBI SRA.

## Abbreviations

aOR: adjusted odds ratio
ASVs: amplicon sequence variants
BV: bacterial vaginosis
CST: Community State Type
FFQ: Food Frequency Questionnaire
HC: hormonal contraception
Molecular-BV: low-*Lactobacillus* vaginal microbiota as characterized in this study by 16S rRNA gene amplicon sequencing
OR: odds ratio
RCT: randomized controlled trial
SD: standard deviation
