# Supplementary material for: Associations between dietary micronutrient intake and molecular-Bacterial Vaginosis
Source: Reprod Health. 2019 Oct 22;16:151. doi: 10.1186/s12978-019-0814-6 (PMC6806504; doi:10.1186/s12978-019-0814-6)
Supplement: Supplementary file 1 — Additional file 1: Table S1. Literature Review: Micronutrients and Vaginal Dysbiosis. Table S2. Relative abundance of major taxa in each CST [file 12978_2019_814_MOESM1_ESM.docx]

**Supplementary Table 1. Literature Review: Micronutrients and Vaginal Dysbiosis**

| **First Author, Year** | **Study Design** | **Study Location** | **Study Population** | **Vaginal Dysbiosis Measure** | **Nutrient measure** | **Study Results** | **Comments** |
| --- | --- | --- | --- | --- | --- | --- | --- |
| **Vitamin D** | | | | | | | |
| Moore 2018(8) | Cohort | Detroit MI | N=1459 African American women, aged 23-34, enrolled in NIEHS study | Self-reported diagnosis of BV by physician between enrollment and first follow-up at 20 mos. | Serum 25(OH)D measured at enrollment.  Modelled continuously on log2 scale or as binary variable based on cut point of 20ng/mL | >16% reported at least 1 BV diagnosis.  >73% were vitamin D deficient  >A doubling of vitamin D was associated with **increased** risk of BV (aRR 1.22 (CI 1.02-1.48), or recurrent BV (aRR 1.30 (CI 0.86-1.36), controlling for age, education, marital status, BMI and alcohol consumption.  >These relationships were similar when vitamin D was modeled as a binary variable. | Self-report of BV. |
| Akoh, 2018(9) | Cohort | Rochester NY | N=158 pregnant women aged 13-18 | Clinical dx of BV during pregnancy (chart abstraction) | Serum 1,25(OH)_2_D measured at mid gestation and delivery | >Teens with 25(OH)D<30ng/mL at mid-gestation more likely to have a diagnosis of BV (p=0.02), however those who tested positive for BV at any point during gestation had higher mean concentrations of 1,25(OH)_2_D at delivery | Did not control some possible confounders e.g. number of sexual partners |
| Turner 2016(11) | Cohort | Zimbabwe | N=571 women participating in the HC and risk of HIV acquisition study who were not on HC | Nugent score | Serum 25(OH)D, in a binary variable (<=30 (insufficient) vs. >30ng/ml (sufficient)) | Insufficient vitamin D at enrollment was NOT associated with increased BV prevalence or incidence, adjusting for age, education, parity, HSV2 status, partner circumcision status, number male sexual partners and intravaginal hygiene practices. | Not an RCT. |
| Taheri 2015(12) | RCT | Tehran, Iran | N=211 women aged 18-35 with asymptomatic BV and vitamin D deficiency (serum <75nmol/l) were given 2000IU/ day of vitamin D3 or placebo | Nugent score and vaginal pH | Serum vitamin D3 | Oral vitamin D was associated with an increased cure rate (OR 10.1, p<0.001) as compared to placebo | Unclear significance of asymptomatic BV |
| Klebanoff 2014(14) | Cohort | Birmingham AL | N=2337 women, mean age 24.7, 88% African American, enrolled in the LVSF study, and seen at least once in each season. | Nugent score | Season as a proxy for vitamin D. | >BV prevalence was 40% in winter, 38% in spring, and 41% in summer and fall.  >Season was not associated with BV in women who were BV-negative at study entry (odds ratio versus winter were 1.0 for spring, 1.0 for summer and 0.9 for fall, p=0.81).  >Among women BV-positive at study entry, the corresponding odds ratios were 0.9, 1.4 and 1.4 (p<0.001). | No measured Vitamin D levels |
| Turner 2014(13) | RCT | Ohio | N=118 women mean age 26, 74% African American with symptomatic BV, intervention group (n=59) received 50,000IU of vitamin D3 over 24 weeks, controls (n=59) received placebo. Both received po. metronidazole. | BV at baseline assessed via Amsel’s criteria.  Recurrent BV assessed via Nugent score at 4, 12, 24 weeks | Serum 25(OH)D | >71% of intervention and 68% of control women were vitamin D deficient at enrollment  >Vitamin D intervention did not improve BV recurrence at any time point | Significant loss to follow-up. |
| French 2011(17) | Cohort | Chicago and New York | N=475 women, 353 HIV+ and 122 HIV- enrolled in WIHS | BV defined by Amsel’s criteria | Serum vitamin D determined by LC-MS/MS,  vitamin D deficiency defined as <=20, insufficiency as >20-<=30, and sufficiency as >30ng/ml | >19% of women (17.3 HIV- and 25.4 HIV+ had BV.  >In HIV+ only: vitamin D deficiency (aOR 3.12, p=0.02) was associated with higher risk of BV, adjusting for race, sex partners in last 6 months, age, smoking alcohol consumption, education, menopause, CD4 count, log HIV RNA. | Not RCT |
| Dunlop 2011(18) | Cohort | Tennessee | N=160 pregnant women from the Nashville Birth Cohort | Nugent | Serum vitamin D, Folate, Omega 6 and Omega 3 | > Adjusted odds of BV were significantly increased among women with BV with 25 (OH) D<12 and folate<5. |  |
| Hensel 2011(16) | Cohort | United States | N=3523 pregnant and non-pregnant women enrolled in NHANES | Nugent | Serum vitamin D and cotinine | >Among pregnant women only, vitamin D deficiency was associated with BV (aOR 2.87, CI 1.13-7.28) |  |
| Bodnar 2009(20) | Cohort | Pittsburgh PA | N=469 pregnant women, 70.9% age 20-29 | BV identified by pH>=4.7 and Nugent score 7-10 | Serum 25(OH)D | >After adjustment for race and STIs, there was increased prevalence of BV associated with serum vitamin D concentrations below 20 and 50nmol/L (lower concentrations)  >In subgroup analysis, there was a linear dose response relationship btw 25(OH)D and the prevalence of BV among black women before and after confounder adjustment. This was not seen in white women. However, there were relatively few white women 17/209 with severe vitamin D deficiency (<20nmol/L). |  |
| **Other Micronutrients: Iron, Vitamin A, C, E, β-Carotene, Folate, Calcium, Diet Indices** | | | | | | | |
| Brabin 2017(10) | Prospective study nested within RCT. | Burkina Faso | N=1954 African women | Nugent score, also 16S rRNA gene sequencing with clustering into CST IV (i.e. “molecular BV”) vs other CSTs. | Iron supplementation | >Prevalence of BV by Nugent or CST IV did not differ between those who received Iron and those who did not.  >At baseline those who were iron deficient were more likely to have normal microbiota.  >Iron was likely poorly absorbed. Systemic iron markers did not change in the supplementation group. | Did not control for confounders.  Systemic iron markers were not significantly changed. |
| Thoma 2011(15) | Cohort | Birmingham AL | N=1735 women, mean age 25.3, 86% African American, enrolled in the LVSF study | Nugent | Diet assessed with Block 98 FFQ, based on this calculated mean daily GI, mean daily GL, HEI and NNR | > GL was positively and NNR negatively associated with BV, after adjusting for age, education, race, cigarette and alcohol use, number of sex partners since previous visit, douching frequency, HC and BMI.  >HEI values >70 were associated with a significant reduction in BV.  >In prospective analysis, GL was associated with BV persistence and progression after adjustment. | No serum measurements |
| Christian 2011(19) | Cohort nested within RCT | Bangladesh | N=1812 pregnant women were randomized to placebo, vitamin A supplementation, or β-carotene supplementation | Nugent score | Supplemented | >Decreased prevalence and incidence of BV at 3 months post-partum in both the vitamin A supplementation and β-carotene supplementation groups. |  |
| Neggers 2007(22) | Cohort | Birmingham AL | N=1827 women, mean age 25.0, 86% African American, enrolled in LVSF study | Nugent  BV: score 7-10,  “Severe BV”: Score >=9, pH>=5 | Diet assessed with Block 98 FFQ, which was used to calculate estimated intake of various dietary factors. | > In adjusted analysis total energy intake was marginally associated with BV,  >Increased total fat intake was significantly associated with BV  >Increased total fat, saturated fat, and monounsaturated fat were significantly associated with severe BV.  >Vitamin E, Folate, and Calcium intake were inversely assoc with severe BV | No serum measurements |
| Tohill 2007(21) | Cross sectional | US (Boston, Providence, New York, Detroit) | N=553 women, 369 HIV+ and 184 HIV-, | Nugent score 7-10 | Serum vitamin C, E, α− and β-carotene, lycopene, β-cryptoxanthin, lutein/zeaxanthin, vitamin A, vitamin B12, folate, ferritin, iron, total iron binding capacity, selenium, zinc | >Higher concentrations of vitamin A, C, E, and β-carotene associated with decreased risk of BV, controlling for confounders including education, *Candida* colonization and frequency of vaginal sex. |  |
| Verstraelen 2005(23) | Nested case control | Belgium | N=115 pregnant women | Nugent score | Serum ferritin, soluble transferrin receptors, CRP | >Based on the log10 soluble transferrin receptor/ferritin ratio, women with subclinical iron deficiency more likely to have BV. | Small sample size, unclear significance of subclinical iron deficiency |
| Belec 2002(24) | Cross sectional | Central African Republic | N=275 women | Nugent score | Serum vitamin A | >Only 15 were considered deficient in vitamin A.  >Vitamin A deficiency was “associated in an additive fashion with the number of genital tract infections diagnosed or seropositivity for active syphilis.” | Small numbers, no direct association with BV |

NIEHS=National Institute of Environmental Health Sciences Study of Environment, Lifestyle and Fibroids, WIHS: Women’s Inter-Agency Health Study, LVSF=Longitudinal study of vaginal flora, BV=Bacterial Vaginosis

**Supplementary Table 2: Relative abundance of major taxa in each CST**

|  |  |  | Relative abundances | | | |
| --- | --- | --- | --- | --- | --- | --- |
| CST | N | Organism | min | median | mean | max |
| I | 45 | L. crispatus | 0.604 | 0.955 | 0.987 | 0.996 |
| II | 2 | L. gasseri | 0.749 | 0.775 | 0.775 | 0.800 |
| III | 30 | L. iners | 0.283 | 0.742 | 0.710 | 0.996 |
| IV | 23 | Gardnerella vaginalis | 0 | 0.219 | 0.205 | 0.461 |
|  |  | Atopobium vaginae | 0 | 0.097 | 0.088 | 0.275 |
| V | 1 | L. jensenii |  |  |  | 0.9613 |

CST=Community State Type, N=total number of samples in a particular CST

**Suppl. Methods: Literature search**

We searched the Pubmed for the terms: ("Micronutrients"[Majr] OR "Dietary Supplements"[Majr] OR "Vitamins"[Majr] OR "Vitamin D"[Mesh] OR "Vitamin A"[Mesh] OR "beta Carotene"[Mesh] OR "Zinc"[Mesh] OR "Nutritional Status"[Majr] OR "Ascorbic Acid"[Mesh] OR "Anemia, Iron-Deficiency"[Mesh] OR "folic acid"[mh] OR “nutritional biomarkers”[tw] OR vitamin*[tw] OR micronutrients[tw] OR “dietary indices”[tw] OR “vitamin a”[tw] OR “beta carotene”[tw] OR “vitamin D”[tw] OR iron[tw] OR zinc[tw] OR folate[tw] OR “fatty acid”[tw] OR “fatty acids”[tw] OR “nutritional status”[tw] OR “dietary supplements”[tw] OR “dietary supplementation”[tw]) AND ("Vaginosis, Bacterial"[Majr] OR “bacterial vaginosis”[tw] OR “vaginal microbiota”[tw] OR “vaginal microbiome”[tw]), through December 2018. The search resulted in 254 articles, which we narrowed based on relevance. References for each article were also searched. Articles not relating micronutrients to BV or vaginal microbiota were excluded, as were articles describing probiotic interventions. (See **Suppl. Table 1 for results**)
