# Supplementary material for: Associations between dietary micronutrient intake and molecular-Bacterial Vaginosis
Source: Reprod Health. 2019 Oct 22;16:151. doi: 10.1186/s12978-019-0814-6 (PMC6806504; doi:10.1186/s12978-019-0814-6)
Supplement: Supplementary file 2 — Additional file 2: Figure S1. List of Main Diet Variables Produced by the Block Brief Questionnaire. [file 12978_2019_814_MOESM2_ESM.pdf]

NutritionQuest

Block Brief 2000 FFQ ©

DIET ANALYSIS OUTPUT VARIABLES

DT_KCAL	CALORIES (Kcal)
DT_PROT	PROTEIN (g)
DT_TFAT	TOTAL FAT (g)
DT_CARB	CARBOHYDRATE (g)
DT_CALC	CALCIUM (mg)
DT_PHOS	PHOSPHORUS (mg)
DT_IRON	IRON (mg)
DT_SODI	SODIUM (mg)
DT_POTA	POTASSIUM (mg)
DT_A_IU	VITAMIN A (IU)
DT_A_RE	VITAMIN A (RE)
DT_THIA	THIAMIN (B1) (mg)
DT_RIBO	RIBOFLAVIN (B2) (mg)
DT_NIAC	NIACIN (mg)
DT_VITC	VITAMIN C (mg)
DT_SFAT	SATURATED FAT (g)
DT_MFAT	MONOUNSATURATED FAT (g)
DT_PFAT	POLYUNSATURATED FAT (g)
DT_CHOL	CHOLESTEROL (mg)
DT_FIBE	FIBER Total dietary fiber (g)
DT_VitE	VITAMIN E a-TE
DT_ZINC	ZINC (mg)
DT_AN_ZN	ANIMAL ZINC, Zinc from animal sources (mg)
DT_VITB6	VITAMIN B6 (mg)
DT_MAGN	MAGNESIUM (mg)
DT_ACARO	ALPHA-CAROTENE (ug)
DT_BCARO	BETA-CAROTENE (ug)
DT_CRYPT	CRYPTOXANTHIN (carotenoid) (ug)
DT_LUTZE	LUTEIN (carotenoid) (ug)
DT_LYCO	LYCOPENE (carotenoid) (ug)
DT_Ret	RETINOL (preformed Vit. A, ug)
DT_ProA	CAROTENE Provitamin A carotenoids (ug)
Genisten	GENISTEIN Genistein (ug)
Daidzen	DAIDZEIN Daidzein (ug)
DT_caffn	CAFFEINE (mg)
DT_vitK	VITAMIN K (ug)
DT_VB12	VITAMIN B12 (ug)
DT_CYSTEN	CYSTEINE (mg)
DT_METHI	METHIONINE (mg)

MeatIron	Iron from Meat (mg)
DT_HEME	Heme iron (mg)
GL	Glycemic Load (glucose), average daily
GI	Glycemic Index (glucose), average daily
DT_FOLFD	Total folate (natural + synthetic), mcg
DT_FOLAC	Folic acid, from food fortification, mcg'
DT_FDFOL	Naturally occurring folate in food, mcg
FOL_DFE	DFE, Average daily Dietary Folate Equivalents, mcg
DT_VITD	Dietary vitamin D, (IU)
DT_SEL	Selenium, mcg
DT_TRFAT	Trans fats, total, gms
DT_SUG_T	Sugars, total, gms
DT_ARGININE	Dietary arginine, mg
DT_COPP	Copper, mg
DT_FA182	Dietary PUFA (~N-6) 18:2, gms
DT_FA183	Dietary PUFA (~N-3) 18:3, gms
DT_FA184	Dietary PUFA (~N-3) 18:4, gms
DT_FA204	Dietary PUFA (~N-6) 20:4, gms
DT_FA205	Dietary N-3 PUFA 20:5 (EPA), gms
DT_FA225	Dietary N-3 PUFA 22:5 (DPA), gms
DT_FA226	Dietary N-3 PUFA 22:6 (DHA), gms
DT_TOTN6	Omega-6 FA, gms
DT_TOTN3	Omega-3 FA, gms
DT_ALCO	alcohol (ethanol), gms
DT_THEO	Theobromine,
Betaine	Betaine, mg
Tcholine	Total choline, mg

GROUP_SOLID_COUNT	# of solid foods respondent reported ever eating
GROUP_SOLID_TOTAL_FREQUENCY	Frequency of all solid foods
GROUP_SOLID_TOTAL_GRAMS	Grams of solid food (g) PER DAY
GROUP_SUGARYBEVG_TOTAL_GRAMS	Sugary beverages, gms
GROUP_SUGARYBEVG_TOTAL_KCAL	Kilocalories from sugary beverages

Percent of Calories

PCTFAT	% of Kcal from fat
PCTPROT	% of Kcal from protein
PCTCARB	% of Kcal from carbohydrate
PCTSWEET	% of Kcal from sweets, desserts
PCTALCH	% of Kcal from alcoholic beverages

Percent of Calories, excluding alcoholic beverages calories from denominator

BA_PFAT	% fat cals, alcoholic beverages excluded from denominator
BA_PPROT	% prot cals, alcoholic beverages excluded from denominator
BA_PCARB	% carb cals, alcoholic beverages excluded from denominator

Fiber from different sources

GROUP_BEANFIBER_TOTAL_FIBE	Fiber from beans (g)
GROUP_VEGETABLESFRUITFIBER_TOTAL_FIBE	Fiber from veg & fruit (g)
GROUP_GRAINFIBER_TOTAL_FIBE	Dietary fiber from grains (g)

FOOD GROUPS (Approximations of Pyramid food groups, pre-2006 serving sizes)

VEGSRV	Daily servings of vegetables
FRUITSRV	Daily frequency of fruits & fruit juices
GRAINSRV	Daily svgs breads, cereals, rice, pasta
MEATSRV	Daily svgs meat, fish, poultry, beans, eggs
DAIRYSRV	Daily servings of milk, yogurt, cheese
FATSRV	Daily svgs fats & oils, sweets, sodas

Nutrients from vitamin supplements

SUP_VITA	Average daily Vit A from supplements (IU)
SUP_VITC	Vit C (mg)
SUP_VITD	Vit D (IU)
SUP_VITE	Vit E (a-TE)
SUP_IRON	IRON (mg)
SUP_CA	CALCIUM (mg)
SUP_ZINC	ZINC (mg)
SUP_BCAR	beta-car (ug)
SUP_B1	B1 (mg)
SUP_B6	B6 (mg)
SUP_B12	B12 (ug)
SUP_FOL	FOLATE (mcg)
SUP_CU	COPPER (mg)
SUP_SE	SELENIUM (mcg)
SUP_B2	B2 (mg)
SUP_MG	MAGNESIUM (mg)
SUP_NIAC	NIACIN (mg)
